# Recent Reports on Polysaccharide-Based Materials for Drug Delivery

**DOI:** 10.3390/polym14194189

**Published:** 2022-10-06

**Authors:** Joanna Kurczewska

**Affiliations:** Faculty of Chemistry, Adam Mickiewicz University, 61-614 Poznan, Poland; asiaw@amu.edu.pl; Tel.: +48-618-291-565

**Keywords:** polysaccharide, alginate, chitosan, hyaluronic acid, pectin, dextran, starch, cellulose, drug delivery

## Abstract

Polysaccharides constitute one of the most important families of biopolymers. Natural polysaccharide-based drug delivery systems are of constant interest to the scientific community due to their unique properties: biocompatibility, non-toxicity, biodegradability, and high availability. These promising biomaterials protect sensitive active agents and provide their controlled release in targeted sites. The application of natural polysaccharides as drug delivery systems is also intensively developed by Polish scientists. The present review focuses on case studies from the last few years authored or co-authored by research centers in Poland. A particular emphasis was placed on the diversity of the formulations in terms of the active substance carried, the drug delivery route, the composition of the material, and its preparation method.

## 1. Introduction

**Figure 1:**
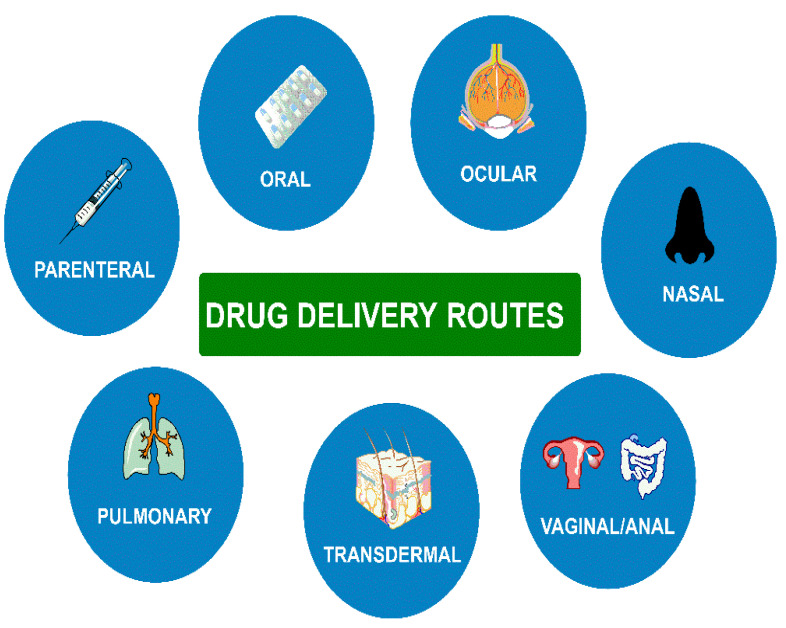
Schematic representation of drug delivery routes.

The breakthroughs of the last century, including the discovery of drugs essential to save lives, contributed to a spectacular improvement in healthcare. Nevertheless, the scientific community conducts ongoing studies in the search for new drugs and improving the effectiveness of those available on the medical market. Many molecules with therapeutic properties that could be used in treating specific diseases or their action could be based on, for example, lower dosages, or encounter numerous barriers in the human body that limit or disqualify their use. One of the directions to improve the therapeutic effects of drugs is the use of drug delivery systems (DDSs) [1,2,3]. DDS not only enables the introduction of therapeutic molecules into the body but also increases safety and efficacy by controlling the rate and place of its release. Therapeutic agents can be delivered by various routes, i.e., nasal, ocular, oral, parenteral, pulmonary, transdermal, and vaginal or anal (Figure 1). Similarly, drug delivery systems are designed to be delivered via various routes. DDSs can also be categorized according to the type of drug carrier materials. DDSs include dendrimers, liposomes, hydrogels, micelles, quantum dots, nanomaterials, and mesoporous or polymeric systems [4]. Despite the application of synthetic and natural polymers, the latter are biocompatible, biodegradable, and not hazardous to the environment. Polysaccharides are an important group of natural polymers found in plants, algae, animals, or microbes [5,6,7].

Polysaccharides (PLS) are composed of monosaccharide units connected via glycosidic bonds, which are susceptible to enzymatic hydrolysis. Their complex structure, including various functional groups, enables chemical modification to give new desired properties. These carbohydrate molecules of high-molecular-weight generally have more than 100 monosaccharide units, and their number can even exceed 100,000. Their structure is linear or branched, but the latter may take the form of short branches with a different distribution on a linear backbone or a branch-on-branch structure [8]. Another classification is based on their charge, and we can distinguish neutral (e.g., dextran), cationic (e.g., chitosan), and anionic (e.g., hyaluronic acid) polysaccharides. The structures of selected polysaccharides are included in Table 1.

The number of documents describing polysaccharides as DDSs is very large and constantly increasing [9]. The polysaccharide-based DDSs can be divided into three general groups: (a) polysaccharide-drug conjugate through interactions between drug and PLS (electrostatic and covalent interaction, direct linkage), (b) drug-loaded particles, and (c) PLS-based hydrogels [5,10]. Although the number of valuable reviews concerning different aspects of biomedical applications of PLS is impressive, it is interesting to analyze the state of research conducted by Polish scientists in this area. In Poland, polymer chemistry is an area of research with a long tradition; hence many research centers can boast of significant achievements and global recognition. In this article, the author will focus on research studies regarding different PLS-based formulations for drug delivery from the past five years presented in original research articles authored or co-authored by scientists with affiliations from Poland. In addition, a brief reference to the latest research directions in this area will be provided to illustrate current trends in polysaccharide-based DDSs. Such a comparison may help determine the level of research conducted in Polish centers concerning the global ones. The present review is limited to a few selected polysaccharides, i.e., alginate, chitosan, hyaluronic acid, pectin, dextran, starch, and cellulose, which attract the most attention in the discussed group of researchers.

## 2. Alginate

Alginates are anionic hydrophilic polysaccharides extracted mainly from brown algae [11] and some soil bacteria. These linear polysaccharides consist of alternating residues of 1–4 α-L-guluronic (G-blocks) and β-D-mannuronic acid (M-blocks). The composition and sequence of G and M blocks can differ (GG, MM, MG, and GM), affecting the material’s molecular weight and physical properties. Thus, the molecular weight of alginate (ALG) varies from 10 to 1000 kDa. The commercial market offers sodium, potassium, and ammonium salts of 60–70 kDa molecular weight. Monovalent ions can be easily replaced with divalent cations (mainly calcium). The strong interactions between calcium ions and carboxyl groups in the polysaccharide form a three-dimensional “egg-box” structure. However, crosslinking between alginate and calcium ions occurs with the participation of GG blocks, while MM and MG are responsible for increasing gel flexibility. Therefore, alginate gel formation strictly depends on the percentage of G and M blocks [11,12]. The alginate-based materials can be used in different medical applications, such as wound healing, tissue engineering, or drug delivery in the form of hydrogels, nano-, microparticles, liposomes, or tablets [13,14].

### Alginate-Based Delivery Systems

Alginate-based DDSs attract much attention because of their inherent properties, low cost, and high availability. The variety of systems using alginates in terms of structure, functions, and carried active substances indicate the high susceptibility of this polysaccharide to modifications, which favors its use in various forms. In general, the concentration of alginates in the formulations is in the range of 1–6% (*w*/*v*).

Hydrogel formulation [15,16,17], capable of absorbing many fluids, is often used as a carrier of different active agents. Recently, they have been successfully used, for example, for cancer therapy [18], optic neuropathy [19], or wound healing [20]. Szekalska et al. [21] prepared ALG hydrogel as a carrier of cynaroside with anti-inflammatory and anti-allergic activities. The biological activity of the materials studied was evaluated in vivo. After topical application of cynaroside, the number of T cells, mast cells, and histiocytes in mouse skin suffering from inflammatory and dermatitis was significantly reduced. Interestingly, alginate also generated some positive effects on reducing inflammatory cells. Although cymaroside, a biologically active agent, most significantly influenced the therapeutic effect, the formula of its administration turned out to be very important. Therefore, much better results were obtained for the drug administered as a hydrogel formulation than in its suspension. The topical application of alginate-based materials has been successfully used to treat post-operative wound infections. The replacement of the traditional intravenous form of antibiotic administration is to reduce the side effects caused by high drug concentrations. Local delivery limits the dose of active substances, thus reducing their toxic effects. Gentamicin-loaded ALG membranes were used to prevent and treat wound infection in bone tissue [22]. It was proven that gentamicin delivery was more sustained and prolonged than from collagen membranes. Moreover, ALG membranes demonstrated antimicrobial activity.

**Figure 2:**
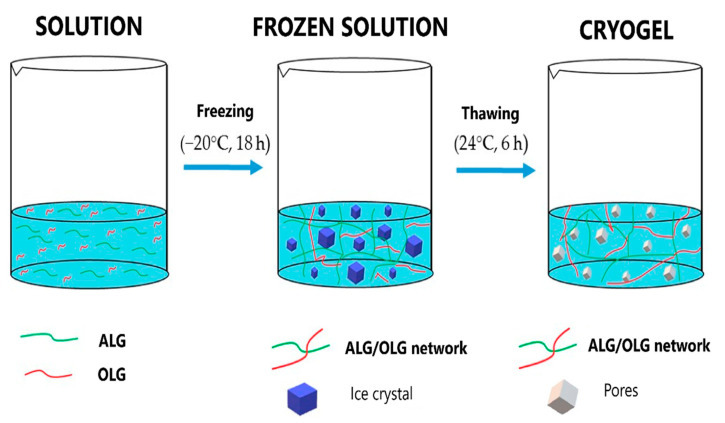
Schematic representation of sodium alginate cryogels preparation by the freeze-thaw method. Reprinted from [24], Marine Drugs 2019.

Importantly, alginate films can not only be dedicated to a topical application but also constitute an alternative to conventional oral formulations and protect a therapeutic agent in the gastrointestinal tract [23]. An antifungal agent—posaconazole—was mixed with sodium alginate and alginate oligosaccharides (OLG), and then the mixture was frozen. Under such conditions, the distance between the polymer chains is reduced, and a crygel is formed after thawing (Figure 2, [24]). The freeze-thaw technique enables much better control of gelling process rate, and the resulting gel is characterized by better mechanical, swelling, and mucoadhesive properties than those obtained with the ionic crosslinking method. Dried crygels poured into plexiglass create mucoadhesive buccal films with prolonged drug release. Additionally, OLG presence increases the antifungal drug activity. The latest scientific reports confirm that the freeze-thaw technique increases the stability and mechanical resistance of alginate-based films [25], and the effective loading of other active substances, e.g., with antioxidant properties [26].

Another interesting application of ALG-based materials was proposed by Karabasz et al. [27,28]. It is part of the current research on using alginates in cancer treatment as a factor in preventing the adverse effects of drugs on healthy cells [29,30]. For this purpose, a natural polyphenol with anticancer activity was entrapped into micelles to increase the drug’s bioavailability. The in vitro studies were promising because the micelles showed high cytotoxicity against different cancer cell lines [27]. However, the in vivo studies gave results slightly below expectations because, despite the non-toxicity of the curcumin carrier, its antitumor activity was limited [28]. Another approach to increasing the availability of curcumin is the use of micro- and macro-particle hydrogel [31]. The preliminary studies showed that curcumin-loaded microparticles demonstrated prolonged drug release and higher cytotoxicity to human colon cancer cells than macroparticles. Notably, the emulsification method can be successfully used to obtain ALG micro-particles for curcumin—a compound with high hydrophobicity. However, water-soluble drugs require other procedures to increase encapsulation efficiency. The alternative strategy is based on the spray-drying method [32]. A one-step drying process was used to encapsulate a model water-soluble drug—metformin hydrochloride—in ALG microparticles crosslinked by calcium chloride. The procedure enables prolonged drug release and improved mucoadhesive properties.

In addition to micro-sized formulations, alginate-based nanomaterials play an increasingly important role in drug delivery. The list of latest reports is extensive and shows great diversity in the alginate-based nanocarriers. Among the recently described nanomaterials, there are e.g., soy protein-ALG nanogels for curcumin delivery [33], inhalable quercetin-alginate nanogel [34], alginate-spermidine micro/nanogels for nerve injury treatment [35] or drug-loaded ALG nanocapsules obtained using different methodologies [36,37]. Concerning this research direction, core-shell alginate nanocapsules with the encapsulated organoselenium compound – ebselen – characterized by antimicrobial activity were prepared by emulsification and gelation with calcium chloride [38]. A hydrophilic ALG shell surrounded the lipophilic drug in the core without covalent interactions confirming the successful separation of core-shell elements. The spherical nanocapsules were investigated as antifungal agents against different pathogenic *Candida* species, and promising results indicated that this could be a novel drug delivery system for therapy in cutaneous candidiasis. Another important group of nanocarriers is nanogels that combine characteristics of large-interface particles, fast phase transition, and the ability of substance encapsulation into their network [39]. Podgórna et al. [40] proposed the application of ALG nanogels in theranostics—a combination of therapy and diagnostics in one package. Gadolinium alginate nanogels were obtained using reverse microemulsion and physical crosslinking. A fluorescent dye—rhodamine B was then encapsulated in the hydrogel network, and finally, the nanocarrier surface was modified by adsorption of polyelectrolyte layers. The material studied demonstrated good stability in time; thus, the toxic effects of free gadolinium ions were not observed. Gadolinium complexes are applied as positive (T1) contrast agents in Magnetic Resonance Imaging (MRI), therefore gadolinium alginate nanogels were visualized by MRI, and the materials studied significantly reduced the relaxation time compared to pure water and analogous calcium alginate nanogels. It confirms their acting as contrast-enhancing agents, hence possible use for theranostic application. As the purpose of ALG nanocarriers can be very diverse, it is also worth analyzing the research conducted by Pathania et al. [41], who prepared nano-herbal drug delivery system. *Phyllantus niruri* is an annual herb with s antibacterial activity against foodborne pathogens. Therefore, its extract can be an alternative to synthetic compounds used in the food industry. As drug carriers in the form of oil-in-water nanoemulsions improve stability and oral bioavailability of lipophilic compounds, it was prepared by low-energy technique in the presence of nonionic surfactant for emulsion stabilization. The formulation improved drug penetration and drug release profile. The plant-based nanoemulsion was active against all the bacteria strains studied because the penetration into bacteria was much easier due to dropletsize below 200 nm. Additionally, the precence of surfactant facilitated crossing lipid barrier in bacterial cells and increased antimicrobial effectiveness. Moreover, it demonstrated satisfactory antioxidant and antifungal properties thus it can be considered as a nanocarrier effective in different research fields.

**Figure 3:**
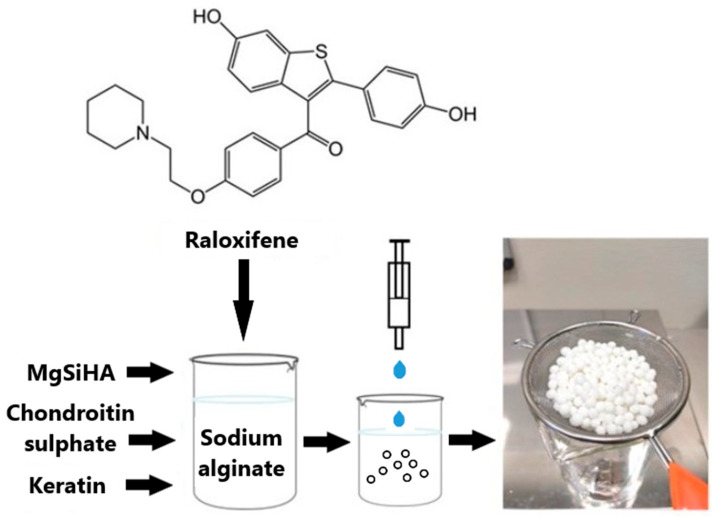
The synthesis procedure of Mg, Si-hydroxyapatite/alginate beads for controlled release of raloxifene. Reprinted from [45], International Journal of Molecular Sciences 2021.

Alginate can also be included in composite drug carriers by combining various materials. Inorganic materials such as silica and clay minerals are often used in such combinations. In this way, nanoparticles of mesoporous silica coated with alginate [42], modified bentonite-alginate nanocomposite gel [43], or montmorillonite-sodium alginate microbeads [44] were lately obtained. Commonly water-insoluble material is suspended in an alginate solution to obtain a multifunctional drug delivery system. Szurkowska et al. [45] combined alginate and hydroxyapatite modified with magnesium and silicon ions (MgSiHA) to obtain composite beads for bone tissue regeneration (Figure 3). In addition to ALG, the organic matrix contained chondroitin sulphate and/or keratin. The material was used as a local carrier of raloxifene hydrochloride to prevent and treat osteoporosis. All the carriers demonstrated satisfactory release profiles of magnesium ions responsible for the biocompatibility of implant materials, as well as the gradual and extended release of silicon ions. Moreover, the materials demonstrated gradual release of raloxifene, but the most favorable profile was obtained with the beads composed of chondroitin and keratin, which could be considered a potential delivery system to bone tissue.

The composite ALG-based materials are often used for transdermal drug delivery, such as microneedles, films, scaffolds, or hydrogels [46]. The latter are often studied for their use as wound dressings. Halloysite, an aluminosilicate clay mineral, is often used as a drug carrier due to its nanotubular structure, biocompatibility, and susceptibility to modification. Incorporating organic functional groups at its surfaces often results in increased drug-carrier interactions. Halloysite functionalized with 3-Aminopropyltrimethoxysilane was successfully applied for loading glycopeptide antibiotic vancomycin and subsequently incorporated into the alginate matrix to obtain a potential wound dressing with antimicrobial activity [47]. The proposed mechanism involved two steps in releasing the drug from the carrier. Initially (up to 6 h), the antibiotic attached to the outer surface of the halloysite is released, while the one enclosed in the lumen is transported into the alginate gel. In the next stage, the drug from the inner surface of the halloysite escapes beyond the matrix, which results in a significant reduction in release rate. The material was characterized by good antibacterial activity and high stability and thus could be applied for long-term treatment of wounds. Such a system could become a universal carrier for drugs of various structures by modifying halloysite surfaces with various reactive functional groups adapted to the guest molecules. Therefore, a similar procedure was repeated to prepare alginate films as food packaging components [48]. Two types of halloysite with different morphology were loaded with salicylic acid and then encapsulated in an alginate matrix. The alginate films demonstrated good antimicrobial activity against food spoilage bacteria and prolonged drug release in the medium mimicking food with liphophilic properties. The latest literature reports confirm the effectiveness of hydrogel prepared by combining halloysite nanotubes with alginate as an excellent carrier for salicylic acid [49].

On the other hand, molecularly imprinted polymers (MIPs), synthesized through copolymerization of functional monomers with crosslinkers, are dedicated to a specific molecule used as a template. Thus vancomycin entrapped inside MIP was encapsulated in an alginate matrix to decrease the antibiotic release rate [50]. In vitro release studies proved that the presence of alginate significantly increased the antibiotic release time. Most of the drug from MIP was released within 12 h, whereas the same amount of vancomycin in MIP/alginate was released after five days while maintaining microbiological activity. Importantly, the effective combination of MIP can apply not only to alginate, but also to other polysaccharides [51,52].

## 3. Chitosan

Chitosan, a copolymer of β(1→4)-2-amino-2-deoxy-D-glucopyranose and β(1→4)-2-acetamido-2-deoxy-D-glucopyranose or homopolymer of β(1→4)-2-amino-2-deoxy-D-glucopyranose, is a natural cationic polymer produced by deacylation of chitin often derived from marine crustaceans, but also insects or microorganisms [53,54,55]. In general, it is characterized by good solubility in acidic media due to the presence of amine groups susceptible to protonation, while it is insoluble in water and alkali. The degree of deacetylation, defined as a mole fraction of free amine groups, is an essential factor influencing chitosan (CS) properties, as only the ones with diacylation above 80% show the highest activity [56]. The commercially available chitosan has a deacylation degree of 70–90%. On the other hand, the percentage of N-acetylated units has an opposite effect because the higher the degree of acetylation, the lower the water solubility and biological activity. The physicochemical and biological properties of chitosan are also strongly dependent on its molecular weight. CS with a molecular weight below 30 kDa can be soluble in water, but the solubility decreases as the weight increases. The polysaccharide of higher molecular weight can be soluble only after protonation of amine groups in the presence of an acidic medium. The presence of amine and hydroxyl functional groups makes it susceptible to modification. Additionally, the biopolymer is biocompatible, non-toxic, and bioadhesive. The crosslinking process of chitosan may proceed through covalent (e.g., glutaraldehyde, epichlorohydrin) or ionic bonds (e.g., citrates, polyphosphates) [57].

The biomedical applications of chitosan-based materials are extensive, ranging from antimicrobial and antifungal activity, gene and drug delivery, anti-HIV and cancer therapies, wound healing, tissue engineering, and many others [55]. The chitosan concentration in the formulations varies depending on the DDS and it can be 0.2–4.0% (*w*/*v*), 4.0–11.0 (*w*/*w*) or 1.0–4.0 (*v*/*v*) [6].

### Chitosan-Based Delivery Systems

**Figure 4:**
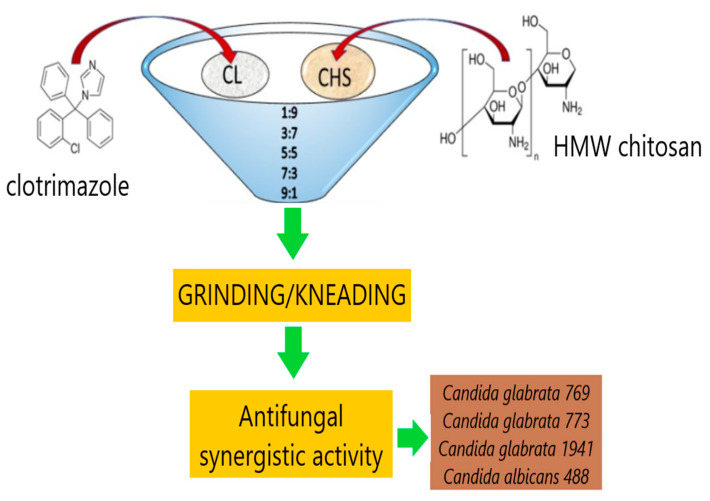
The procedure of preparation and synergistic antifungal effect of high molecular weight chitosan—clotrimazole mixture. Adapted from [58], Marine Drugs 2020.

The CS-based formulations often do not require a sophisticated structure to effectively act as potential carriers of active substances. Grimling et al. [58] investigated a combination of high-molecular-weight (HMW) CS with clotrimazole in a solid powder mixture. Clotrimazole is an imidazole derivative with antifungal activity. The mixtures were prepared using grinding and kneading methods with three samples of high-molecular-weight CS used as an excipient. Chitosan improved the dissolution rate of clotrimazole, increased the effect of drug particle wetting, and thus prevented its aggregation. The mixture containing chitosan with the highest molecular weight demonstrated a synergistic effect against *Candida* at pH 4. Thus it can be considered an alternative antifungal formulation (Figure 4). Notably, recently published articles still propose simple carriers based on chitosan with antifungal activity, e.g., containing two active substances—nystatin and propolis [59] or caspofungin [60]. Moreover, chemically modified CS was successfully used in the form of N-(2-hydroxy)-propyl-3-trimethylammonium, O-palmitoyl chitosan nanoparticles as a clotrimazole carrier for topical treatment [61], while poloxamer modified CS nanoparticles for acyclovir delivery [62].

In addition to synthetic drugs with antifungal properties products of natural origin are often used [63,64]. *Chelidonium majus* is an isoquinoline alakloids-rich plant. Its extracts can be used to treat fungal or bacterial skin infections. Some of their isolated alkaloids could find application in trichomoniasis of the vagina. Therefore, Paczkowska et al. [65] designed a CS delivery system with *Chelidonii herba* extract for vaginitis treatment. The advantage of *C. herba* extract is its multifunctional action, as it is not dedicated to the specific microbe but demonstrates antibacterial, antifungal, and antiviral activities. The system was prepared by mixing lyophilized extract of *C. herba* with chitosan and adding different excipients to obtain mucoadhesive vaginal tablets. The combination of the active components of plant extract and chitosan showed a synergistic effect. Therefore, the tablets studied can be considered a potentially safe and effective formulation for treating bacterial and fungal vaginal infections. A similar carrier, chitosan-gel containing *Mitracarpus frigidus* extract has been recently proposed for vulvovaginal candidiasis treatment [66].

Besides simple CS-based formulations prepared by mixing solid components, most of such carriers are obtained in solutions. For example, lipid-chitosan nanoparticles were applied for entrapment and controlled delivery of cisplatin to tumor cells [67]. Low-molecular-weight CS was dissolved in acetic acid. Then, the drug was introduced into it. The aqueous solution was combined with an ethanolic lipid solution to obtain nanoparticles by ionic gelation. A lipid layer contributed to a significant increase in the encapsulation efficiency of the poorly water-soluble cisplatin compared to simple polymeric nanoparticles. The drug release rate was controlled by a CS matrix, while the lipid layer prevented its leakage.

In general, CS nanoparticles are found interesting due to the excellent solubility of hydrophobic drugs, their permeability through biological membranes, and improved transfer of active agents to the target area. Among the world’s latest reports there are e.g., injectable cisplatin-loaded CS nanoparticles [68] or N-2-hydroxypropyl trimethyl ammonium chloride chitosan and N,O-carboxymethyl chitosan nanoparticles with encapsulated amoxicillin [69]. An interesting application for CS nanocarriers has been proposed by Dharshini et al. [70]. pH-sensitive CS nanoparticles were loaded with the anti-HIV drug dolutegravir. The drug is orally administrated once daily, which is challenging in pediatric patients due to the necessity of swallowing. The proposed formulation assumes adding an appropriate drug dose with milk or porridge as a food admixture. CS, isolated from novel crab species, was characterized by lower molecular weight and a higher degree of deacylation than the commercial biopolymer. The nanoparticles in powder form were prepared using the spray drying technique. The stability and dissolution rate of dolutegravir was significantly improved after encapsulation in the CS nanoparticles. Additionally, the proposed nanoformulation as food admixture should facilitate administration to very young patients, while maintaining the therapeutic effect.

DDSs of controlled particle sizes include not only nanosystems but also micro-materials [71,72,73]. The microcrystalline chitosan-based 3D formulation was used for the controlled delivery of meloxicam, a non-steroidal anti-inflammatory drug for treating complications after tooth extraction [56]. The microcrystalline CS is characterized by better bioactivity and sorption than chitosan, while the formulation could be applied as an effective dental dressing. A different use for CS microparticles was demonstrated by Szymańska et al. [74]. A water-soluble chitosan derivative, CS glutamate, was mixed with antiretroviral drug–zidovudine–solution to obtain mucoadhesive microparticles for vaginal applications. In general, mucoadhesive DDSs initially contact the mucous membrane and swell, while mucoadhesive compounds are activated due to moisture presence. Such formulation could be dedicated for local drug delivery to buccal, nasal, or vaginal cavities. Bartkowiak et al. [75] applied several mucoadhesive commercial polymers, including chitosan, as matrices of fluconazole in the form of tablets and studied the drug release from simulated saliva and vaginal fluids.

On the other hand, Szymańska et al. [76] focused on the effect of unmodified chitosan and beta-glycerophosphate-crosslinked CS on mucoadhesive, rheological, and drug release properties of the CS hydrogels containing clotrimazole as an active agent upon storage. The long-term studies showed poor stability of unmodified CS matrix, while beta-glycerophosphate stabilized the hydrogel, primarily upon storage in the refrigerator. The proposed modification of chitosan also had a beneficial effect on drug content stability, which was observed up to 6 months of storage. These findings could be significant in designing formulations for chronic wound treatment, requiring sustained long-term therapeutic effects. Chanaj-Kaczmarek et al. [77] used chitosan as a matrix for a hydrogel delivery system containing *Calandule flos* lyophilized extract with determined concentrations of active components—chlorogenic acid and narcissin. *Calandule flos* can reduce inflammation and wound healing, but it requires bioadhesive additives to apply to chronic wounds. In the optimized proportions, the hydrogels demonstrated controlled release of the active components, antimicrobial and anti-hyaluronidase activities.

Some research centers focus on more sophisticated CS-based potential drug carriers, which are intended to increase the effectiveness of the designed materials. Piegat et al. [78] proposed combining 3D printing with electrofluidodynamic methods for synthesizing hierarchical multilayered scaffolds. CS grafted with linoleic acid produced electrosprayed spheres uniformly distributed on struts and nanofibers. The preliminary results indicate the possibility of their use in tissue engineering and drug delivery. Another innovative approach is presented by Janus et al. [79], who obtained chitosan-based quantum dots for biomedical applications. Quantum dots (QD), the nanoparticles with luminescent properties, could find application in medicine or pharmacy. However, they must meet the requirements of biocompatibility and non-cytotoxicity to be considered for such a purpose. The literature reports demonstrate different approaches to connecting quantum dots with chitosan through, e.g., CS covalent attachment to graphene quantum dots [80], CS coating of graphene QD combined with magnetite [81], or graphene oxide QD functionalized with CS nanoparticles and polyethylene glycol [82]. In order to prepare the material according to Green Chemistry, carbon chitosan-based quantum dots were prepared using microwave radiation and functionalized with some amino acids (e.g., lysine, cysteine). The nanoparticles demonstrated good photoluminescence properties and a lack of cytotoxicity. Another new material, hybrid theranostic cubosomes, was described by Bazylińska et al. [83]. These cubic liquid-nanocrystalline nanoparticles were stabilized with a multilayer shell composed of chitosan, a single strand of DNA, and folic acid-chitosan conjugate. The nanocarrier studied was designed as a potential medium for anticancer therapy, as it included up-converting Er^3+^, Yb^3+^ codoped NaYF_4_ as energy harvester and diagnostic agent, daunorubicin as photosensitive anticancer drug and DNA as model material for gene therapy. As it demonstrated multifunctional properties, the cubosomes could be used for the photodynamic treatment of tumor lesions. Finally, an interesting literature report on the research into improving the mechanical properties of CS-based carriers was also presented by Wang et al. [84]. Electrospun nanofibers of polyacrylonitrile, cellulose acetate, and silica were introduced into chitosan hydrogel using homogenous dispersion and lyophilization to obtain a three-dimensional matrix without chemical crosslinking. The material exhibited improved mechanical properties and the capability of a bioactive factor immobilization, thus showing great potential for tissue engineering.

Many research studies concentrate on composite materials, including chitosan and other components such as biological species [85,86], metals [87,88,89], synthetic polymers [90,91,92], and others. A foam complex between negatively charged DNA and cationic chitosan was studied as a functional material for drug delivery [93]. Appropriate composition ratios can control the physicochemical and mechanical properties of DNA-CS scaffolds in order to obtain suitable materials for biomedical applications. Interestingly, many papers describe the attachment of inorganic units to chitosan to obtain materials of advanced properties. Chitosan-gold hybrid nanoparticles represent an example of such a sophisticated nanocarrier [94]. Gold nanoparticles of limited antibacterial activity were combined with CS and used as nanocarriers of *Punicagrantum* L. extract. The active agent of natural origin demonstrates high antibacterial, antiviral and antifungal activities. Therefore it can be applied for infection treatment caused by antibiotic-resistant bacteria. The nanocomposite showed a synergetic effect against methicillin-resistant bacteria strain—*Staphylococcus aureus*—and high stability at different pH conditions. A similar system, CS-Au nanoparticles in polyvinyl alcohol nanofiber mats, was applied for the topical delivery of *Punica grantum* L. extract [95]. Glutaraldehyde-crosslinked polyvinyl alcohol nanofibers as a platform for incorporating the composite CS-Au nanoparticles with entrapped plant extract demonstrated enhanced mechanical properties, long-term stability, sustained release of an active agent, and high antibacterial activity.

**Figure 5:**
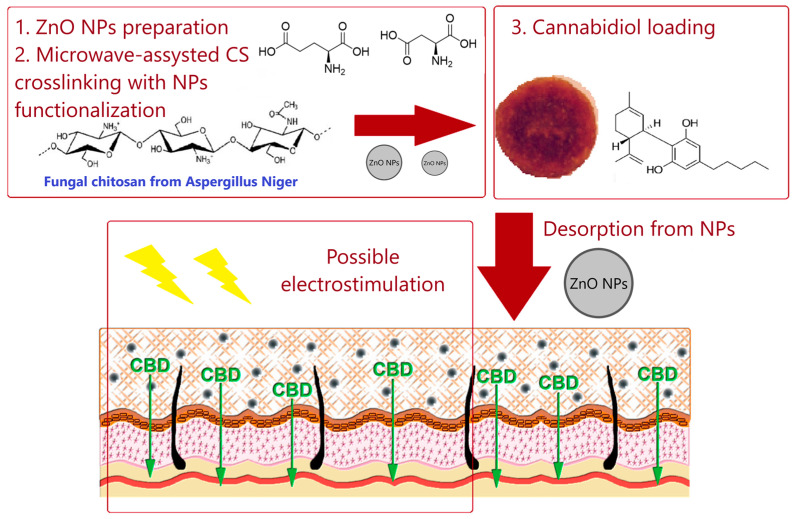
Schematic representation of preparation and application of CS-ZnO nanoparticles for cannabidiol delivery. Reprinted from [96], Polymers 2021.

On the other hand, CS nanoparticles with ZnO could be considered effective transdermal systems [96]. The composite material was applied for controlled delivery of a natural product—cannabidiol—and could be used to treat drug-resistant epilepsy (Figure 5). Cannabidiol, a poorly water-soluble compound, has limited bioavailability after oral administration. Thus, transdermal delivery is considered a suitable alternative. The hydrogel studied was prepared using fungal chitosan and various amounts of ZnO nanoparticles. The addition of zinc oxide resulted in enhanced mechanical durability, drug loading capacity, and prolonged release. The nanocarrier studied was characterized by conductive properties that could be applied in iontophoresis. Therefore, it could be considered a stimuli-responsive system placed on the skin. The same research group used these promising results to generate transdermal DDS. Chitosan crosslinked with azelaic acid was functionalized with zinc oxide nanorods to obtain a transdermal system with encapsulated acetylsalicylic acid for long-term use [97]. The active component can be administered through skin tissue bypassing the digestive tract, effectively preventing cardiovascular diseases.

Zinc-modified CS nanoparticles can also be applied in cancer therapy. For this purpose, chitosan was used as a nanocarrier of doxorubicin and then stabilized by Zn^2+^ incorporation into the CS backbone [98]. The presence of zinc ions resulted in better stability, drug binding capacity, and low toxicity of CS nanocarrier. Another interesting vehicle for doxorubicin is furcellaran/chitosan nanocapsules [99]. Furcellaran is a sulfated polysaccharide and, similarly to chitosan, belongs to natural polyelectrolytes. The nanocapsules were prepared as multilayers using electrostatic interactions between two biopolymers. In order to obtain a nanocarrier that delivers a drug to a targeted site, the nanocapsules were modified using homing peptide. The results proved excellent selectivity for treating malignant cell lines without harming nonmalignant ones.

**Figure 6:**
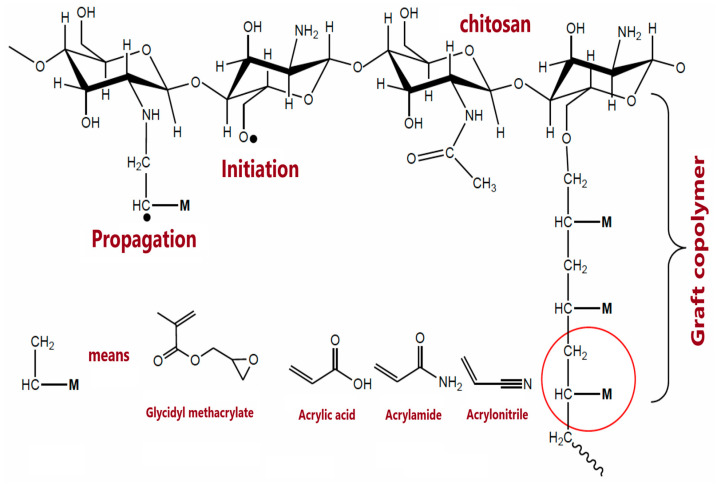
Scheme of binary graft copolymerization. Reprinted from [101], Sensors 2022.

The composite materials often contain other polymers influencing physicochemical properties and drug delivery capabilities. Bil et al. [100] obtained multifunctional microspheres of chitosan incorporated into polyester urethane. CS is responsible for drug delivery, while the crosslinked polyurethane matrix is for shape memory. The material could be considered a minimally invasive surgery system or shape-memory implant with sustained drug release. A different idea is described by Chopra et al. [101], who prepared modified CS hydrogels by graft-copolymerization of two comonomers, acrylamide and acetonitrile (Figure 6). The drug release, diclofenac sodium, was investigated in different conditions showing prolonged release in alkaline media. On the other hand, methacrylic anionic copolymers (Eudragit S-100) were used as coating of CS microspheres to obtain an oral formulation of green tea polyphenon-60 (PP60) with the delayed release [102]. PP60 has a beneficial effect on decreasing oxidative stress or metabolic risk factors, while Eudragit S-100 coating should ensure the active agent release in the ileum.

Many carriers composed of chitosan and other polymers are dedicated to wound healing [103,104,105]. Polyvinyl-CS hydrogels with lignin nanoparticles were synthesized to analyze the influence of lignin on the physicochemical, antibacterial, and antioxidant properties of the composite material [106]. Lignin had a beneficial effect on the porosity and accessibility of water. However, only 1 wt% of these nanoparticles enhanced mechanical and thermal properties while a higher amount agglomerated, limiting access to the hydrogel. The synergistic antioxidative and antibacterial effect of lignin and chitosan indicated increased material effectiveness compared to unmodified CS. Another approach uses poly(chitosan-ester-ether-urethane) hydrogel for genistein release [107]. Genistein is a skin protective agent of low stability; thus, the studied system allowed controlled release with no matrix cytotoxicity and may be considered a potential system in dermatology and cosmetology.

**Figure 7:**
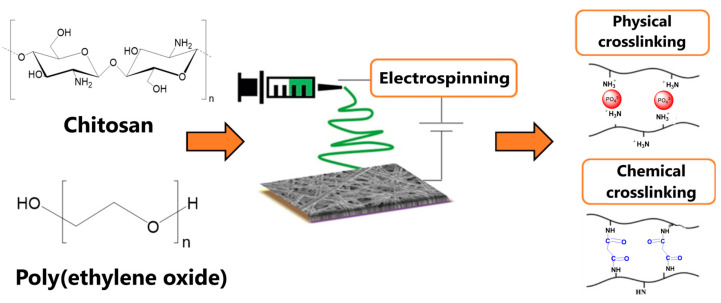
Fabrication of CS-based electrospun membranes. Reprinted from [109], Polymers 2021.

In contrast to active agents with non-specific action, Urbanek et al. [108] demonstrated wound dressing dedicated to a specified pathogenic bacteria—*Staphylococcus aureus*. The nanowovens prepared from poly(lactide-*co*-glycolide) blend with chitosan were functionalized with Auresine *Plus*—an enzyme with antistaphylococcal activity. The enzyme was immobilized through physical adsorption and covalent bonding. Both methods formed materials with good antibacterial activity, while only covalent interactions provided sufficient enzybiotic attachment. The physical or chemical crosslinking of CS-based materials can also significantly influence the mechanical or biological properties of potential wound healing systems. Dodero et al. [109] applied the electrospinning method to prepare CS-based nanofibrous membranes crosslinked by phosphate ions (physically) or ethylene glycol diglycidyl ether (chemically), Figure 7. Physical crosslinking resulted in the fabrication of thinner and homogenous nanofibers of greater porosity, mechanical stability, and water permeability than chemically crosslinked equivalents. Moreover, only the first one was not toxic to cell lines studied.

Additionally, chitosan can also play a complementary role in complex DDSs, where its function is limited to improving selected parameters of the carrier. For example, a liposome formulation coated with CS was used as a medium for intranasal ghrelin administration [110]. The intranasal route is less invasive and painful; hence it is often considered for chronic administrations. The presence of chitosan coating improved ex vivo permeation and mucoadhesive properties.

## 4. Other Polysaccharides

Alginate and chitosan carriers represent the dominant group of DDSs based on polysaccharides recently studied by Polish scientists. Nevertheless, it is worth following the application of other biopolymers from this group.

### 4.1. Hyaluronic Acid

Hyaluronic acid (HA) is a macromolecule of natural origin present in epithelium and neural tissues. It comprises 1,4-β D-glucuronic acid and 1,3-β N-acetyl glucosamine units. Its physiological activities and biocompatibility make it an excellent material for pharmacological applications. On the other hand, poor mechanical stability and fast biodegradation are significant factors that seriously limit its use. Therefore, it is generally combined with other polymers to improve the properties of the carrier [14,111] and applied in different concentrations (0.5–2.0% *w*/*w*, 30–50% *w*/*w*) HA is characterized by high molecular weight, up to 10MDa, and is capable of water binding. Due to its unique properties, it can be used as an active ingredient in formulations, e.g., as an agent with regenerating properties in eye drops [112]. It is often used as a factor to improve the condition of the skin. One of the proposals is to introduce it into the water-in-oil microemulsion as a form of transdermal transport, e.g., in combination with collagen [113,114].

HA is a system susceptible to chemical modifications, which enables adapting its properties to pharmaceutical applications. One of the strategies involves the introduction of long fluorinated aliphatic chains to increase the lipophilicity of the system and, thus, more efficient transport across cell membranes [115]. Moreover, HA can be a precursor for preparing other DDS—porous calcium carbonate [116]. The entrapment of a controllable amount of HA inside CaCO_3_ makes the material promising for biomedical applications.

**Figure 8:**
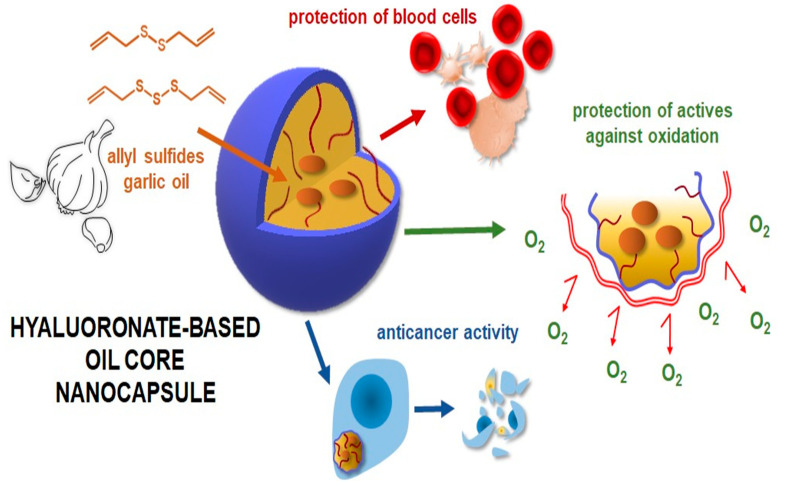
Potential biological activity of HA-based nanocapsules. Reprinted from [122], Nanomaterials 2021.

Drug delivery systems with hyaluronic acid are often in the form of nanocapsules [117,118]. Hydrophobically modified HA by perfluorinated alkyl, and dodecylamine chains served as shells, while corn oil was a nanocapsule core [119]. The nanocarrier was loaded with curcumin for vascular delivery in treating cardiovascular pathologies. Curcumin, as poorly water-soluble and non-selective, requires high doses to show a therapeutic effect, but the nanoformulation enables the drug dose limitation due to direct targeting to the vascular wall and improved bioavailability. Hyaluronate-based shell is a generally promising solution for the delivery of lipophilic compounds. Szafraniec et al. [120] prepared a series of hyaluronates modified by aliphatic amines of different lengths. The most stable capsules contained dodecyl chains and were found as potential vehicles dedicated to a targeted delivery to the liver and lung. Amphiphilic derivatives of HA were used to stabilize oleic acid in core-shell nanocapsules [121]. The materials were compared with analogous CS-based nanocapsules. HA had a crucial role in efficient drug delivery, which was not observed for the CS equivalent. As active agents often show low stability, HA-based nanocapsules can perform a protective function. Encapsulation of garlic oil active components—diallyl disulfide (DADS) and diallyl trisulfide (DATS)—in oil-core nanocapsules was investigated as a potential system for anticancer therapy [122], Figure 8. HA shell protected sulfides against oxidation, inhibited the red blood cell membrane lysis, and limited interactions with digestive truck components. On the other hand, the anticancer activity was maintained in a complex formulation, while the bioavailability of reactive components was improved. The anticancer properties of composite nanocarrier were also obtained using the *Amanita muscaria* mushroom extract as an antitumor agent [123]. The hydrogel was composed of HA as a gel-formating agent, silver and iron oxide nanoparticles, and the extract as a capping agent. The nanoparticles were covered with a layer of compounds present in the extract, which increased the anti-cancer effectiveness, while HA facilitated transport inside the cell lines studied.

HA is sometimes used as an auxiliary compound. An interesting application is presented in the work of Reczyńska et al. [124]. The nanoparticles of poly(DL-lactic-*co*-glycolic-acid) loaded with anticoagulant drug eptifibatide were coated with polyelectrolyte multilayer composed of polycation poly-L-lysine and polyanion HA.

### 4.2. Pectin

An anionic polysaccharide, pectin (PC), is extracted from different plants (e.g., citrus, apple, sugar beet, pumpkin, peach). It is composed of α-(1,4) linked D-galacturonic units. PC’s solubility, viscosity, and gelling ability strictly depend on the degree of methyl esterification. Low methoxy (LM) pectins (<50%) are soluble in water and more stable to pH, moisture, and heat than high methoxy (HM) (>50%) ones. The gelation process requires acidic conditions and sugar for HM PC, while for LM PC—divalent ions. Their low stability limits the application as DDSs, but PC can be combined with other species to increase mechanical strength and decrease degradation [5,111,125]. The concentration of pectin in different formulations varies: 2–4% (*w*/*v*), 4–29% (*w*/*w*).

A good solution for improving PC-based carriers’ properties is adding synthetic polymers [126]. PC mixed with polyacrylic acid was found to be an effective carrier of salicylic acid for potentially treating colon diseases [127]. A similar synthetic procedure was applied to prepare biopolymeric pectin milibeads [128]. The formulation consisted of amidated PC and a synthetic polymer (polyacrylic acid, polyvinylpyrrolidone, polyethylene glycol, or arostoflex) with entrapped drug—5-aminosalicylic acid, active in inflammatory bowel disease treatment. The milibeads were crosslinked using calcium ions. The synthetic polymers incorporated into pectin resulted in prolonged drug release, presumably due to the interactions between natural and synthetic polymers. Additionally, the mechanical strength of composite beads was improved compared to pure PC ones. Further studies on the biopolymeric beads were conducted to investigate the material’s applicability as colon-targeted DDS [129]. The most promising formulation was the one with polyacrylic acid, as it was more stable and capable of overcoming variable conditions of the digestive tract during drug delivery to the colon.

Another approach is to use an inorganic additive for additional functionality. Calcium-rich bioactive glass particles were applied as an inorganic filler to induce pectin crosslinking [130]. The bioactive particles were a source of calcium ions, displayed antibacterial activity, and induced pectin mineralization.

### 4.3. Dextran

Dextran (DX) is a representative of neutral polysaccharides and consists of mainly α-1,6 linked glucopyranoside units and small amounts of α-1,2; α-1,3; and α-1,4 branched chains [131]. Lactic acid bacteria produce it, but commercially available biopolymer comes from sucrose-containing sources. It can be easily functionalized through hydroxyl functional groups. Dextran is biodegradable, biocompatible, hydrophilic, non-toxic, and stable in the bloodstream. It demonstrated antithrombotic and anti-inflammatory activity. It was used (in concentrations below 10% *w*/*w*) to prepare hydrogels [132,133], micelles [134,135,136], or core-shell structures for drug delivery [10,111].

Dextrans with different molecular masses were used to synthesize drug-delivery nanoparticles [137]. Polyaldehydedextran was obtained using sodium mediaperiodate as an oxidizing agent in a different ratio. The nanoparticles composed of 70 kDa DX derivative with a 5% degree of oxidation and 50% substitution of dodecylamine were stable and capable of anticancer drug attachment -doxorubicin. This chemotherapeutic drug could also be entrapped in another formulation, a thermosensitive star-like copolymer [138]. The nanosystem, dextran-graft-poly-N-iso-propylacrylamide, was used for targeted delivery of toxic doxorubicin, and its efficiency was compared to the free drug form. The water-soluble copolymer showed higher toxicity toward cancer cells and required lowered concentration to obtain a therapeutic effect; thus, the formulation is a promising doxorubicin delivery platform.

### 4.4. Starch

Another neutral polysaccharide, starch (ST), is composed of two different chains: linear amylose and branched amylopectin [5,139]. Amylose (20–30% of ST) consists of α-D-glucose units linked via 1,4-glycosidic bonds, while amylopectin (70–80% of ST) is a branched polymer of α-D-glucose units linked via 1,4- and 1,6-glycosidic bonds. In its native form, ST (in varied concentrations: 0.1–0.6% *w*/*w*, 20–30% wt) has limited application as a drug carrier due to low mechanical stability but can be easily modified to improve physicochemical properties. For this reason, literature reports on drug carriers based on pure starch are rare, and only a few articles on this topic can be found, e.g., ST films as carriers for non-steroidal anti-inflammatory drugs [140]. An effective method to improve the properties of starch is an adequately selected cross-linking agent, e.g., maltodextrin [141], or double modification of the biopolymer in the presence of sulfobetaine and deoxycholic acid [142].

An interesting strategy assumes the conjugation of ST-derivative with therapeutics. Hydroxyethyl starch and methotrexate were conjugated and used as a chemotherapeutic for tumor-bearing mice [143]. The nanoconjugate demonstrated greater immunomodulatory and higher tumor growth inhibition than free methotrexate. Therefore, it can be considered a potential anticancer agent in chemo- and chemoimmunotherapy. On the other hand, long-term DDSs for anti-cancer therapy were proposed by Labus et al. [144]. The thermal extrusion process of ST results in the biopolymer destructuring to homogenous melt—thermoplastic starch. The processed ST was combined with polylactide to obtain composite films for prolonged drug delivery of anti-cancer agents.

The biological activity of different agents is used in biomedical applications and food technology for active packaging. The materials protecting against food spoilage bacteria that are safe in contact with food are particularly interesting. Several new ST-based films with incorporated active components demonstrated antibacterial and antioxidant properties. Potato starch-furcellaran-gelatin film was a carrier of lavender oil [145]. Furcellaran is another negatively charged linear polysaccharide derived from red algae with the potential to produce edible films [146]. Gelatin ensures biocompatibility, biodegradability, low cost, and plasticity. ST macrostructure is similar to synthetic polymers, therefore it is the base of the film. Finally, lavender oil demonstrates antioxidant activity and should also improve the functional properties of the composite material. Other proposed biodegradable films included low-density polyethylene, corn starch and nanoclay, and Cloisite 20A [147]. ST was used for partial replacement of synthetic polymer, while nanoclay—was for improving mechanical properties. The active components, Ethylenediaminetetraacetic acid (EDTA) and palm seed extract, had good antibacterial activity against food pathogens but weakened the film’s mechanical properties.

The ST-based carrier could also be effective for encapsulating an active agent—fish oil—characterized by strong odor and poor stability in water. Barley β-D-glucan and waxy maize starch derivative—sodium octenyl succinate, formed a wall, while the cod liver was a core of microcapsules [148]. β-D-glucan is a soluble dietary fiber composed of glucose monomers combined with 1,3- and 1,4-glycosidic bonds. The core-shell structure demonstrated a positive health effect. β-D-glucan decreased the bulk density and oxidation of oil fish, while positively influencing the encapsulation efficiency.

**Figure 9:**
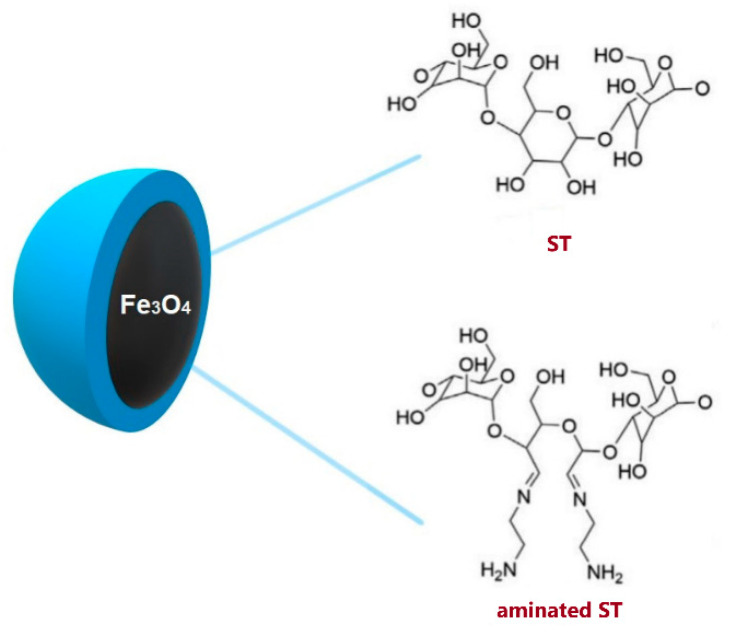
Magnetite nanoparticles coated with ST and aminated ST. Reprinted from [149], International Journal of Molecular Sciences 2021.

Starch can also be used as an effective coating of nanocarriers. Native and aminated ST was applied to coat magnetite nanoparticles, Figure 9 [149]. Fe_3_O_4_ is effective in many areas of medicine but requires modification to prevent oxidation and agglomeration. Reactive amine groups on the surface of aminated ST protected the magnetic core and improved the binding of drugs and biomolecules.

## 5. Complexed Delivery Systems

Drug carriers, which combine the properties of several different polysaccharides, have recently become very popular among Polish scientists. In this type of composite systems, cellulose derivatives are often present.

Cellulose (CL) is derived from plants, algae, fungi, or bacteria and consists of β-1,4 glucose units forming parallel chains. Bacterial CL is very pure and forms small nanofibers, while plant CL has high mechanical strength. Regardless of its source, cellulose has high crystallinity and biocompatibility [139] and is used in different concentrations (2–4% *w*/*v*, 20–50% *w*/*w*). Carboxymethyl cellulose sodium salt is a very popular CL derivative with hydrophilic properties. Tsirigotis-Maniecka et al. [150] proposed a composite hydrogel based on carboxymethyl CL and sodium alginate for enhanced delivery of biflavonoid, hesperidin, with a wide range of biological activities. The properties of the composite hydrogel were compared with hesperidin-loaded alginate and hesperidin-loaded carboxymethyl cellulose particles. The material studied demonstrated improved stability and prolonged delivery of the active component in a more controlled manner.

**Figure 10:**
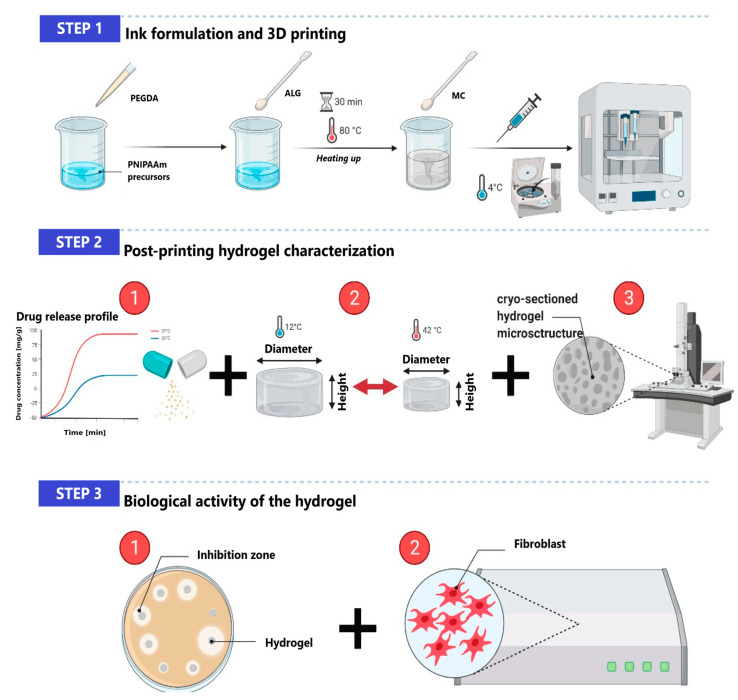
Preparation, characterization, and biological activity of 3-D printed hydrogel. Reprinted from [154], Bioengineering 2021.

Another inexpensive and biocompatible CL derivative is hydroxypropyl cellulose. It was used as a component of bioactive dressing with corticosteroid dexamethasone as an active agent [151]. ALG and hydroxypropyl CL were applied for the drug encapsulation and crosslinked with calcium ions. Then the particles were coated with chitosan and deposited on bionanocellulose sheets modified with carboxyethyl groups. The bioactive material was characterized by prolonged dexamethasone release in a controlled manner, non-toxicity, and anti-inflammatory activity. Therefore it can be considered for the treatment of post-operative wounds. The analogous formulation was used to encapsulate pioglitazone, an antidiabetic drug effective in skin ulcer treatment [152]. Chitosan had a beneficial effect on drug stability inside the particles and prolonged release up to 5 days. The same system was also adapted for curcumin delivery as a potential wound healing dressing [153]. On the other hand, methylcellulose was a component of thermoresponsive 3D-printible hydrogel loaded with antimicrobial mixture of 0.1% *w*/*w* of octenidine dihydrochloride and 2% *w*/*w* of 2-phenoxyethanol [154]. The printable ink contained poly(N-isopropylacrylamide) precursors, sodium alginate, and methylcellulose (Figure 10). Poly(ethylene) glycol was a hydrophilic co-monomer and crosslinker of poly(N-isopropylacrylamide). This temperature-responsive hydrogel showed sustained drug release, antibacterial activity, and non-toxicity. The fabrication of ALG/CL-based delivery systems is constantly developed [155], as evidenced by the work on the application of microfibrillated cellulose-reinforced alginate microbeads for vitamin E delivery [156], cellulose nanofibril/sodium alginate hydrogels for ibuprofen delivery [157] or ALG/hydroxymethyl CL buccal films for allergy treatment [158].

In addition to composite DDSs with cellulose, ALG is also often combined with pectin. The latest literature reports show the effectiveness of alginate/pectin microcapsules for encapsulation and controlled release of anthocyanins [159] or ALG-PC hydrogel films for diabetic wound healing [160]. Therefore, alginate nanoparticles, loaded with the drug, incorporated within pectin microspheres were studied as a potential nasal delivery system of dexamethasone [161]. The dry powder showed moderate swelling and mucoadhesive properties in contact with the simulated nasal fluid. The formulation demonstrated higher stability over liquid one and prolonged drug release. Pectin was also successfully combined with chitosan to prepare multilayer films for buccal administration of clotrimazole [162]. Layer-by-layer deposition of two biopolymers was used to prepare the films, and three different formulations with various distributions of clotrimazole in each layer were studied. The uniform distribution of the drug in PC and CS layers was the most promising for the modified release profile. Clotrimazol from the pectin layer was an initial dose, while the one from the CS layer—demonstrated prolonged release. The material showed antifungal activity additionally enhanced by chitosan. The latest reports confirmed the effectiveness of combining pectin with chitosan, where CS/PC nanoparticles were used as quercitin carriers [163].

Multi-polysaccharide materials obtained with the participation of chitosan are a common practice [164,165,166]. An interesting procedure was described by Gilarska et al. [167], which concerned the combination of chitosan, hyaluronic acid, and collagen for the production of an injectable hydrogel. The hydrogels were chemically crosslinked with gemipicin, a compound of natural origin with additional anti-inflammatory or neuroprotective properties. On the other hand, simple thin films based on collagen, CS, and HA served as carriers for the antibiotic gentamicin [168]. The antibiotic in a complex matrix demonstrated enriched inhibition growth of selected pathogenic Gram-positive and Gram-negative bacterial strains. The same materials’ properties encouraged obtaining chitosan-collagen-hyaluronic acid scaffolds crosslinked by dialdehyde starch. The crosslinking agent improved the mechanical and strength parameters of the material [169]. The scaffolds were then used for in situ calcium phosphate precipitation [170] with a homogenous structure. Both the crosslinker and inorganic additive improved the biocompatibility of the scaffolds. Similar composite materials were obtained with another crosslinking agent—a mixture of N-(3-dimethylamino propyl)-N’-ethylcarbodiimide hydrochloride and N-hydroxysuccinimide with improved stability in aqueous conditions [171].

Among the composite systems studied, those based on chitosan and alginate are also of great importance. Literature data from the last few months indicate their numerous applications including e.g., mucus-penetrating nanoparticles with entrapped berberrine [172], ALG-coated chitosan nanoparticles for protein drugs’ [173] or tamoxifen citrate [174] protection, ALG/CS nanocarriers for co-delivery of doxycycline, florfenicol and silver nanoparticles [175], a nanoparticles-in-microparticles system for curcumin delivery [176], a physically and chemically crosslinked hydrogel composed of CS oligosaccharide and ALG for hydrophobic ketoprofen delivery [177] or CS/ALG microspheres loaded with quercetin [178]. On the other hand, Gierszewska et al. [179] investigated pH-sensitive hydrogel membranes based on CS/ALG polyelectrolyte complex crosslinked by tripolyphosphate. The ionic crosslinking agent influenced molecular structure, swelling properties, and roughness. The material had good swelling/deswelling ability and good pH sensitivity. On the other hand, a very complex system prepared in a three-step procedure was proposed to deliver omega-3-rich oils [180]. The oils with bentonite clay were encapsulated in ALG, CS or combined ALG/CS microspheres. Only the composite microspheres showed the ability to protect the active substance against oxidation and its sustained release. A different approach involves using alginate as a carrier of the active ingredient, surrounded by a second layer of the oppositely charged polymer by electrostatic complexation. For that purpose, negatively charged ALG microparticles were loaded with esculin—a phenolic derivative of plant-origin with therapeutic properties—and further series of positively charged polymers (CS, gelatin, poly(allylamine hydrochloride) or poly(4-styrenesulfonate)) were adsorbed on their surface to form polyelectrolyte shells [181]. In the next stage, the research was extended and both alginate and carboxymethylcellulose were used as a drug carrier, while chitosan or gelatin was used as a coating [182]. Such treatments are beneficial in the rational design of carriers of natural origin substances by controlling several parameters, i.e., size, morphology, encapsulation efficiency, and drug release kinetics, based on selected polymers forming the core and shell.

## 6. Conclusions

This review demonstrates the great potential of a polysaccharide-based drug delivery systems and the significant participation of the Polish scientific community in their development. The unique properties of this group of polymers, i.e., natural origin, biocompatibility, non-toxicity, and susceptibility to modification, contribute to their continued popularity. Table 2 summarizes the selected drug delivery systems described in the present paper. Notably, the research studies of Polish scientists perfectly illustrate world trends in this area. Based on recent reports, it can be noted that the main routes of drug delivery are transdermal and oral. However, polysaccharide-based systems dedicated to other delivery routes are increasingly emerging. The systems containing chitosan or alginate come to the fore, while the remaining ones, probably due to their lower stability, are less frequently studied. The materials effectively deliver therapeutic agents in various formulations—hydrogels, films, capsules, powders, or nanocontainers. Several treatments are used to improve the properties of the carriers. They involve the chemical modification of polysaccharides, cross-linking, and grafting, or the formation of complex materials. Such composite supports are obtained by covering the core with an active substance with a protective shell or by slightly more sophisticated methods involving adding other components. Inorganic materials, synthetic polymers, or a blend of polysaccharides have been used successfully in this role. Such treatments allow not only the protection of a sensitive active substance but also its controlled release in a targeted area. In many cases, this reduces the drug dose, thus eliminating its side effects. It is crucial in cancer therapy and the treatment of chronic diseases.

Despite extensive research in this area, using polysaccharides as drug delivery systems has its limitations. The low stability of the carrier and the rapid release of the active agent greatly limit the practical use of unmodified biopolymers. Physical cross-linking is often insufficient, while chemical one requires the introduction of organic compounds, which should be avoided in biomedical applications. The composite materials, including an inorganic matrix of natural origin, significantly improve the properties of such systems. On the other hand, the biodegradability of inorganic materials remains unclear. Moreover, very sophisticated procedures for obtaining the optimal carrier can generate high costs and thus eliminate the material from practical use. Therefore, future research should concentrate on the properties of formulations in terms of optimized drug encapsulation and targeted release, the influence of other components and additives, and cost estimation.

Additionally, most of the research focuses on demonstrating the synthesized systems’ improved properties, adequate active substance protection, controlled release in the appropriate dose, healing abilities, stability, and non-toxicity of the formulations. Unfortunately, the research showing the interaction of these DDSs with the human body is limited, while such knowledge is necessary to transfer them from the laboratory scale to the industry dedicated to biomedical materials. Therefore, further advanced research in this direction needs to be developed.

## Figures and Tables

**Table 1:** Structures and properties of selected polysaccharides.

Name	Structure	Charge	Origin
Alginate (ALG)	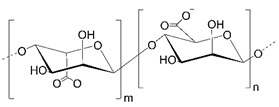	negative	algae
Chitosan (CS)	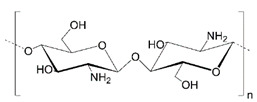	positive	animals
Hyaluronic acid (HA)	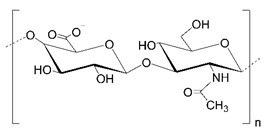	negative	animals
Pectin (PC)	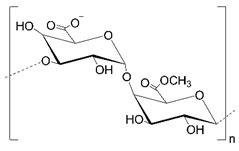	negative	plants
Dextran (DX)	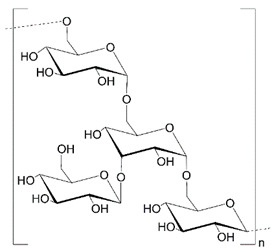	neutral	microorganisms
Starch (ST)	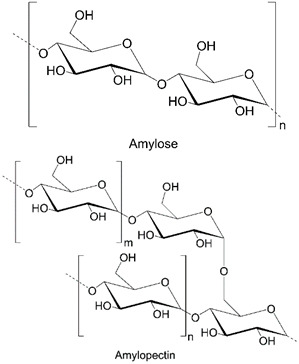	neutral	plants
Cellulose (CL)	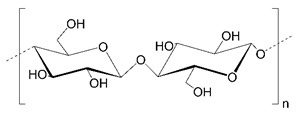	neutral *	plants

* derived from cellulose hydroxypropylcelluloses, hydroxypropylmethylcelluloses, methylcelluloses—neutral, carboxymethylcelluloses—negative.

**Table 2:** Delivery routes of the selected polysaccharide-based DDSs.

Delivery Route	Polysaccharide	Formulation	Active Agent	Reference
oral	ALG	films	posaconazole	[24]
	ALG	micelles	murcumin	[27]
	ALG	nanogels	gadolinium	[40]
	CS	nanoparticles	cisplatin	[67]
	CS	nanoparticles	doxorubicin	[98]
	CS	microspheres	polyphenon-60	[102]
	HA	nanocapsules	garlic oil	[122]
	PC	milibeads	mesalazine	[128]
	ST	films	doxorubicin	[144]
	CL, ALG	hydrogel	hesperidin	[150]
	CS, PC	films	clotrimazole	[162]
	CS, HA	nanocapsules	oleic acid	[121]
	CS, ALG	microspheres	omega-3 oil	[180]
ocular	HA	drops	choline salicylate	[112]
nasal	ALG, PC	powder	dexamethasone	[161]
vaginal/anal	CS	powder	Chelidonii H.	[65]
	CS	microparticles	Zidovudine	[74]
	CS	tablets	fluconazole	[75]
transdermal	ALG	hydrogel	cymaroside	[21]
	ALG	membrane	gentamicin	[22]
	ALG	nanocapsules	ebselen	[38]
	CS	powder	clotrimazole	[58]
	CS	hydrogel	Calendulae flos	[77]
	CS	nanoparticles	cannabidiol	[96]
	CS	nanorods	azelaic acid	[97]
	CS	hydrogel	genistein	[107]
	CL, ALG, CS	microparticles	dexamethasone	[151]
	CL, ALG	hydrogel	2-phenoxyethanol	[154]
	CS, HA	films	gentamicin	[168]
pulmonary	DX	powder	cromoglycate	[183]
parenteral	PC	hydrogel	bioactive glass	[130]
	CS, HA	hydrogel	genipin	[167]

## Data Availability

Not applicable.
